# Human monocytes differentiate into tumor-associated macrophages upon SKOV3 cells coculture and/or lysophosphatidic acid stimulation

**DOI:** 10.1186/s12950-022-00307-w

**Published:** 2022-07-16

**Authors:** Ying Feng, Meizhu Xiao, Guangming Cao, Hao Liu, Yanfang Li, Shuzhen Wang, Stan Zijtveld, Bert Delvoux, Sofia Xanthoulea, Andrea Romano, Chongdong Liu, Zhenyu Zhang

**Affiliations:** 1 Department of Obstetrics and Gynecology, Beijing Chao-Yang Hospital, Capital Medical University, North Road of Workers Stadium, No. 8Chaoyang District, Beijing, 100020 China; 2 Department of Obstetrics and Gynecology, GROW-School for Oncology and Developmental Biology, Maastricht University Medical Centre, Maastricht, The Netherlands

**Keywords:** Serous ovarian cancer, Monocytes, Tumor-associated macrophages, LPA, SKOV3

## Abstract

**Background:**

Serous ovarian carcinoma is the most common type of ovarian carcinoma. Tumor-associated macrophages (TAMs) promote ovarian cancer progression. Most macrophages are generated by monocyte differentiation. Lysophosphatidic acid (LPA) levels are high in blood, tissues and ascites of patients with ovarian cancer. This study investigated whether human monocytes can directly differentiate into TAMs in the serous ovarian carcinoma microenvironment.

**Methods:**

Human monocytes were isolated and purified from umbilical cord blood. A serous ovarian carcinoma-like microenvironment was generated by coculturing monocytes and SKOV3 cells in 0.4-μm-pore-size Transwell chambers. Additionally, the effect of LPA was assessed. The two cultured cell types and supernatants were evaluated.

**Results:**

The morphology and function of monocytes cocultured with SKOV3 cells and/or stimulated with LPA were significantly changed compared with those of non-stimulated monocytes. The CD14 + CD163 + and CD206 + phenotype indicated that stimulated cells were TAMs. The induced cells promoted SKOV3 cell proliferation and invasion, further proving that they were TAMs. The level of the cytokine interleukin-6R in the supernatant was significantly elevated in the treatment groups compared to the control monocyte group. Pathway enrichment analysis of ELISA results showed a strong influence of interleukin-6 family signaling, especially the JAK-STAT signaling pathway, further confirming the importance of IL-6R.

**Conclusion:**

Monocytes can differentiate into TAMs under coculture with SKOV3 cells and/or LPA stimulation. The induced TAMs promote SKOV3 cell proliferation and invasion. The cytokine receptor IL-6sR and the JAK-STAT signaling pathway play an important role in the differentiation of monocytes into TAMs.

## Background

Ovarian carcinoma is the leading cause of death among gynecological cancers and the fifth leading cause of malignancy-related death in women  [1, 2]. The prognosis of ovarian cancer is poor because most cases have metastasized before diagnosis and are not diagnosed until they are in an advanced stage [3]. In 2021, an estimated 21,750 new cases of ovarian carcinoma will be diagnosed and 13,770 deaths from ovarian cancer will occur in the USA [1]. Global new cases and deaths for ovarian cancer were 313,959 and 207,252 in 2020 [4]. The 1-year, 5-year and 10-year survival rates are approximately 72%, 48% and 35%, respectively [5, 6]. Serous ovarian cancer is the most common pathological type of ovarian cancer, and is the main focus of this study.

The unique tumor microenvironment (TME) of ovarian cancer promotes tumor metastasis, immunosuppression and drug resistance [7]. Tumor-associated macrophages (TAMs) are important.

components of the TME and play considerable roles in all aspects of ovarian cancer [8]. TAMs modulate the effectiveness of various antitumor treatments [9–14]. They usually show an M2-like phenotype, and ovarian cancer TAMs are reported to express high levels of CD163 and CD206 [15].

The survival and prognosis of ovarian cancer patients are strongly associated with TAMs, especially M2-like (CD14 + CD163 +) TAMs [16]. CD163 and CD206 function as immunosuppressive receptor molecules, and their expression usually indicates early recurrence and decreased relapse-free survival (RFS) times in patients with ovarian cancer [15, 17–19].

In humans, macrophages are derived from the differentiation of monocytes [20–22]. Most macrophages at disease sites arise from the polarization of circulating monocytes [22]. Monocytes are attracted by chemokines, enter sites of damage and differentiate into macrophages. Monocytes can supplement the population of resident macrophages by differentiating into macrophages and can respond to inflammatory signals at disease sites under different conditions [20–22]. Therefore, the TAMs in serous ovarian cancer may also be derived from circulating monocytes.

Various mediators can affect the activation and function of TAMs [8]. Lysophosphatidic acid (LPA), a potential marker for ovarian cancer initially called ovarian cancer growth factor and ovarian cancer activator, enhances tumorigenesis and ascites formation [23–26]. LPA levels are significantly increased in the blood, tissues and ascites of patients with epithelial ovarian cancer [27]; the pathological concentration is 2.0–22 μmol/L, and the LPA levels have been reported to be related to the formation and malignant behavior of ovarian cancer stem cells [28, 29]. Moreover, LPA regulates macrophage polarization [30]. One study reported that LPA can induce murine monocytes to differentiate into macrophages that express high levels of CD206, but CD163 expression was not assessed in that study [30].

Based on the above background information, the following hypothesis was proposed: human monocytes can directly differentiate into TAMs in the serous ovarian cancer TME where the LPA level is high. Our article proposing this hypothesis has been published [31].

In this study, we further explored the role of LPA and tumor cells in the differentiation of TAMs. To this end, a coculture system of primary monocytes and SKOV3 serous ovarian carcinoma cells was established to simulate the in vivo microenvironment of serous ovarian cancer. We used a 0.4-μm pore size Transwell where monocytes and SKOV3 cells were cultured in distinct chambers in medium either with or without LPA. Whereas cells cannot pass through the membrane, the exchange of cytokines is not affected. Our study had three goals based on the use of this system. First, we explored the effects of coculture with SKOV3 cells and/or LPA stimulation on monocytes. Second, we explored the effects of coculture with monocytes and/or LPA stimulation on SKOV3 cells. Third, we explored the changes in cytokines in the medium.

## Results

### Morphology and function of induced monocyte-derived cells under the conditions of coculture with SKOV3 cells and/or LPA stimulation were changed significantly compared to those of monocytes

**Fig. 1:**
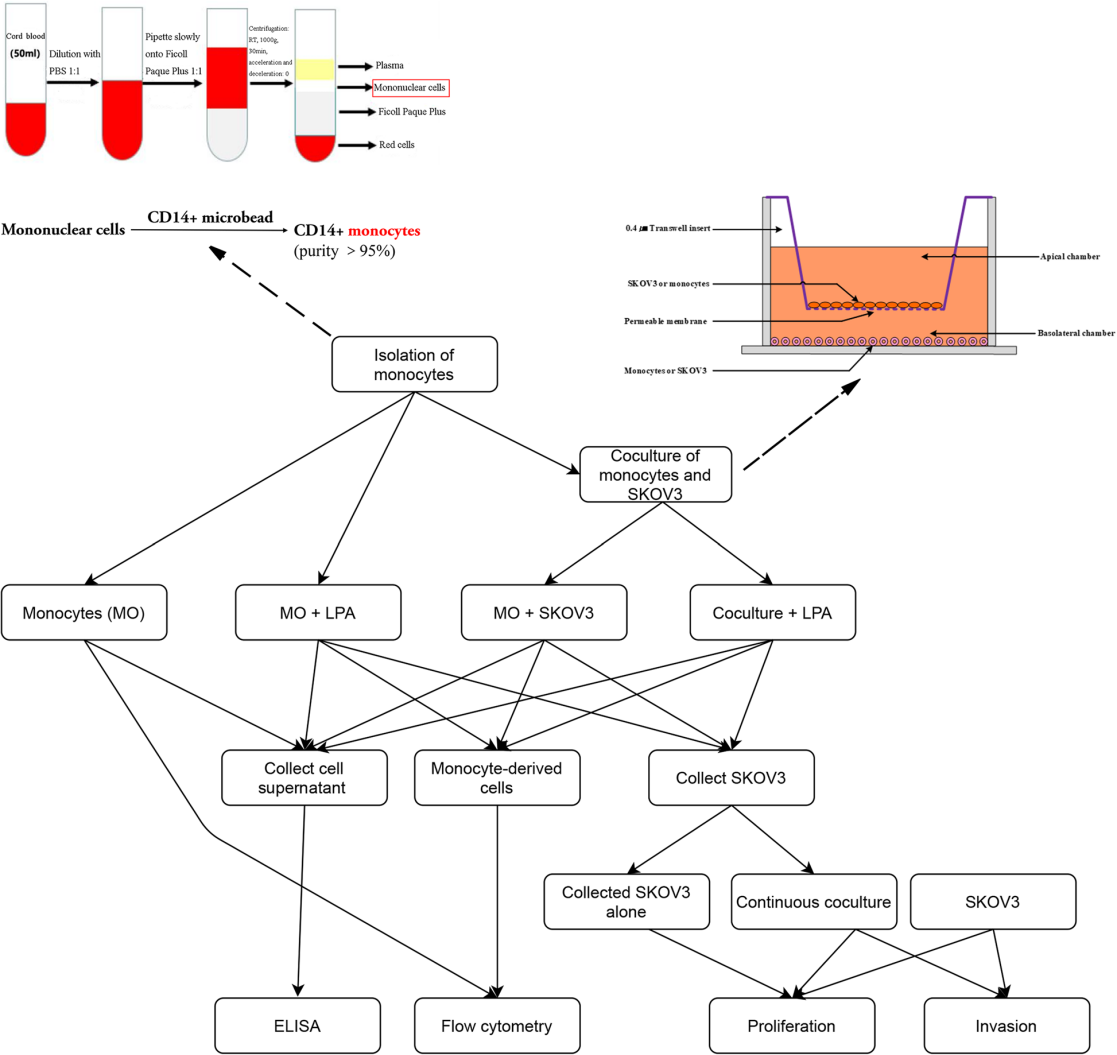
The experimental flowchart of this study

**Fig. 2:**
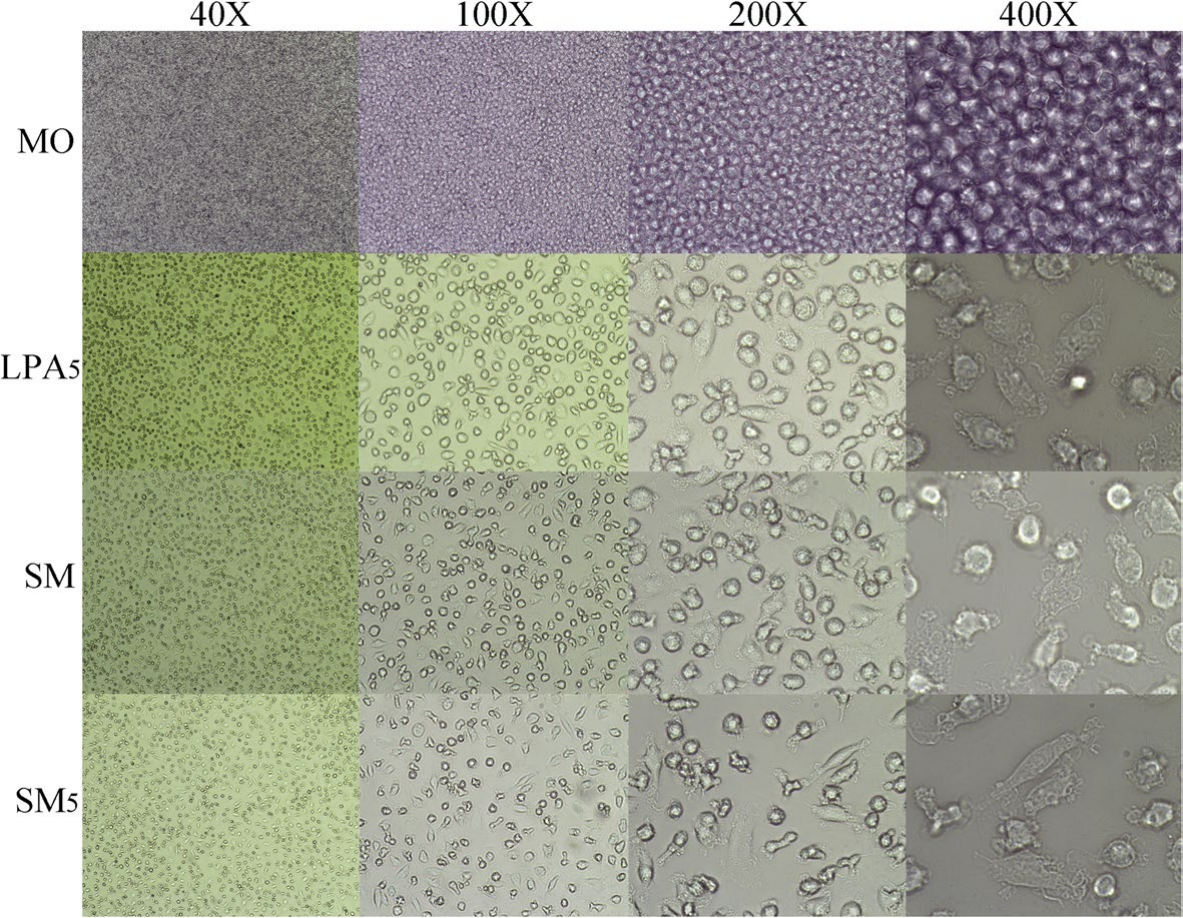
The morphology of induced monocyte-derived cells under the conditions of coculture with SKOV3 cells and/or LPA stimulation was changed significantly compared to those of monocytes. Monocyte-derived cells cocultured with SKOV3 cells and/or stimulated with LPA (LPA5, SM and SM5) showed adherent cell growth, an increased size, an irregular shape, pseudopodia and protrusions. Notes: 1) MO: monocytes were cultured alone in LPA-free medium; 2) LPA5: monocytes were cultured alone in medium with 5 μM LPA; 3) SM: monocytes were cocultured with SKOV3 cells in LPA-free medium; and 4) SM5: monocytes were cocultured with SKOV3 cells in medium with 5 μM LPA

After 7 days, as shown in Fig. 1, the morphology of monocyte-derived cells in the 3 treatment groups (LPA5, SM and SM5) changed significantly compared to that of cells in the MO group. The MO group contained cells growing in suspension and with a small, round and regular shape. The other 3 treatment groups contained adherent cells with an increased size, and morphological diversity. Pseudopodia and protrusions were also observed in these groups. The irregular shapes indicate the active functions (Fig. 2).

**Fig. 3:**
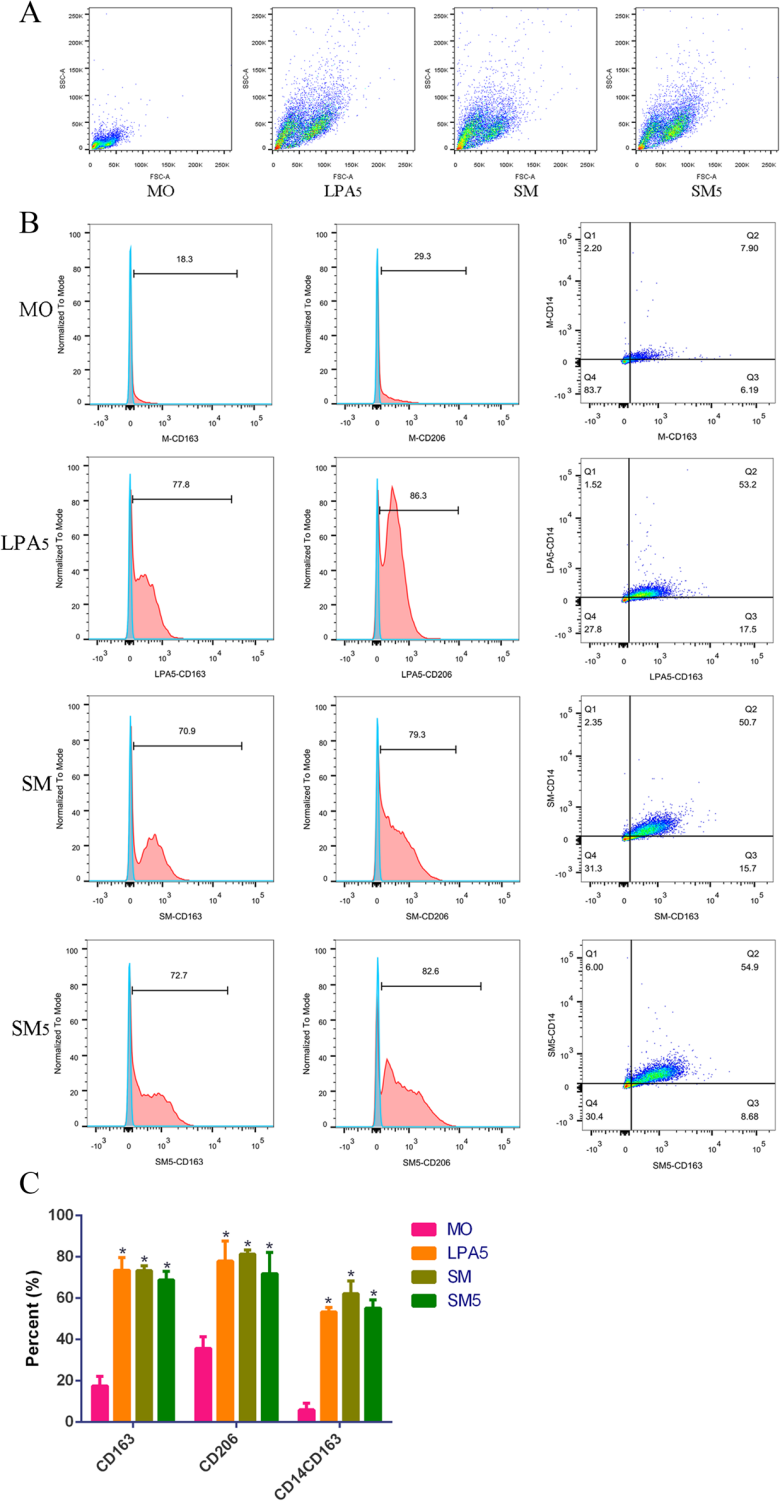
The monocytes differentiated into CD163 + macrophages under the conditions of coculture with SKOV3 cells and/or LPA stimulation. **A** The distribution of cells under the same voltage: monocyte-derived cells in the 3 treatment groups (LPA5, SM and SM5) were much larger and more dispersed than those in the MO group. **B**, **C** Monocyte-derived cells in the 3 treatment groups (LPA5, SM and SM5) showed positive expression of CD163 and CD206 and coexpression of CD14 and CD163 (CD163 + , CD206 + , CD14 + CD163 +). Notes: *, *p* < 0.0001 compared to monocytes

These cells were subsequently analyzed by flow cytometry after 7 days of treatment. The distribution under the same voltage showed that monocyte-derived cells in the 3 treatment groups (LPA5, SM and SM5) were much larger and more dispersed than those in the MO group (Fig. 3A). Moreover, monocyte-derived cells in the 3 treatment groups (LPA5, SM and SM5) showed positive expression of CD163 and CD206 and co-expression of CD14 and CD163 (CD163 + , CD206 + , CD14 + CD163 +) (Fig. 3B). Statistical analysis of the percentage of positive cells revealed significant differences between each of the 3 treatment groups (LPA5, SM and SM5) and the MO group (*p* < 0.0001, Fig. 3C). However, there was no difference among the 3 treatment groups (LPA5, SM and SM5).

These results indicated that monocytes differentiated into CD163 + macrophages under the 3 tested conditions (LPA5, SM and SM5).

### The induced monocyte-derived cells promoted the proliferation and invasion of SKOV3 cells, further proving that these cells were TAMs

**Fig. 4:**
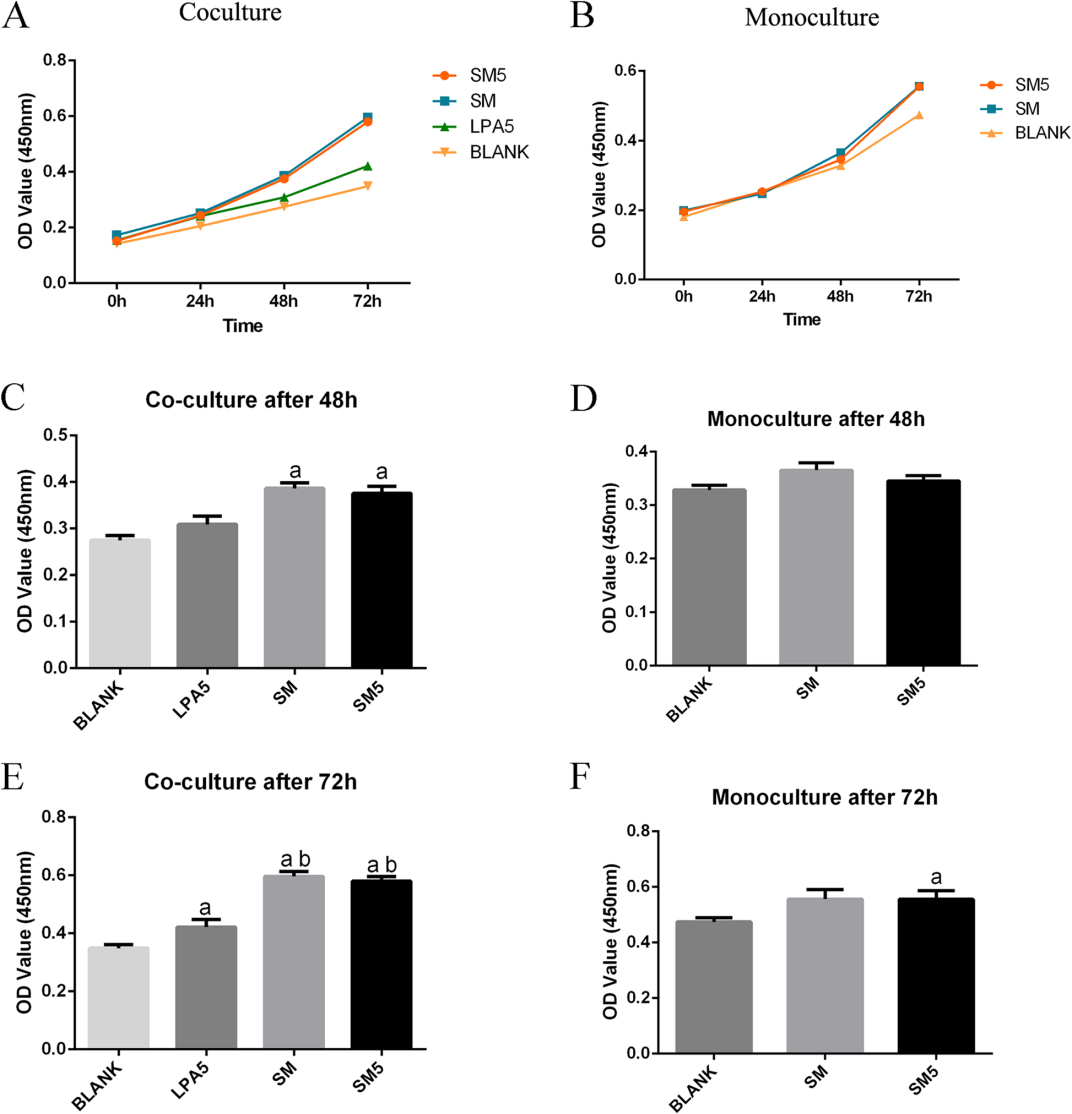
The induced monocyte-derived cells promoted the proliferation of SKOV3 cells. Under continuous coculture conditions (**A**, **C**, **E**), the number of SKOV3 cells in the coculture groups increased significantly beginning on the second day. A difference was observed under monoculture conditions (**B**, **D**, **F**) beginning on the third day. Notes: Coculture (A, C, E): continuous coculture of cells in the SM group and SM5 group after 7 days of treatment. Monoculture (**B**, **D**, **F**): SKOV3 cells in the SM group and SM5 group were cultured alone after 7 days of treatment. a, *p* < 0.05 compared to blank; b, *p* < 0.05 compared to LPA5

Two different conditions (coculture and monoculture) were established for the proliferation assay. “Coculture” indicates continuous coculture of cells in the SM group and SM5 group after 7 days of treatment. “Monoculture” indicates that SKOV3 cells in the SM group and SM5 group were cultured alone after 7 days of treatment. Under continuous coculture conditions, as shown in Fig. 4, the number of SKOV3 cells in the coculture groups increased significantly beginning on the second day. A difference was observed under monoculture conditions beginning on the third day. This coculture environment greatly influences tumor cell proliferation.

**Fig. 5:**
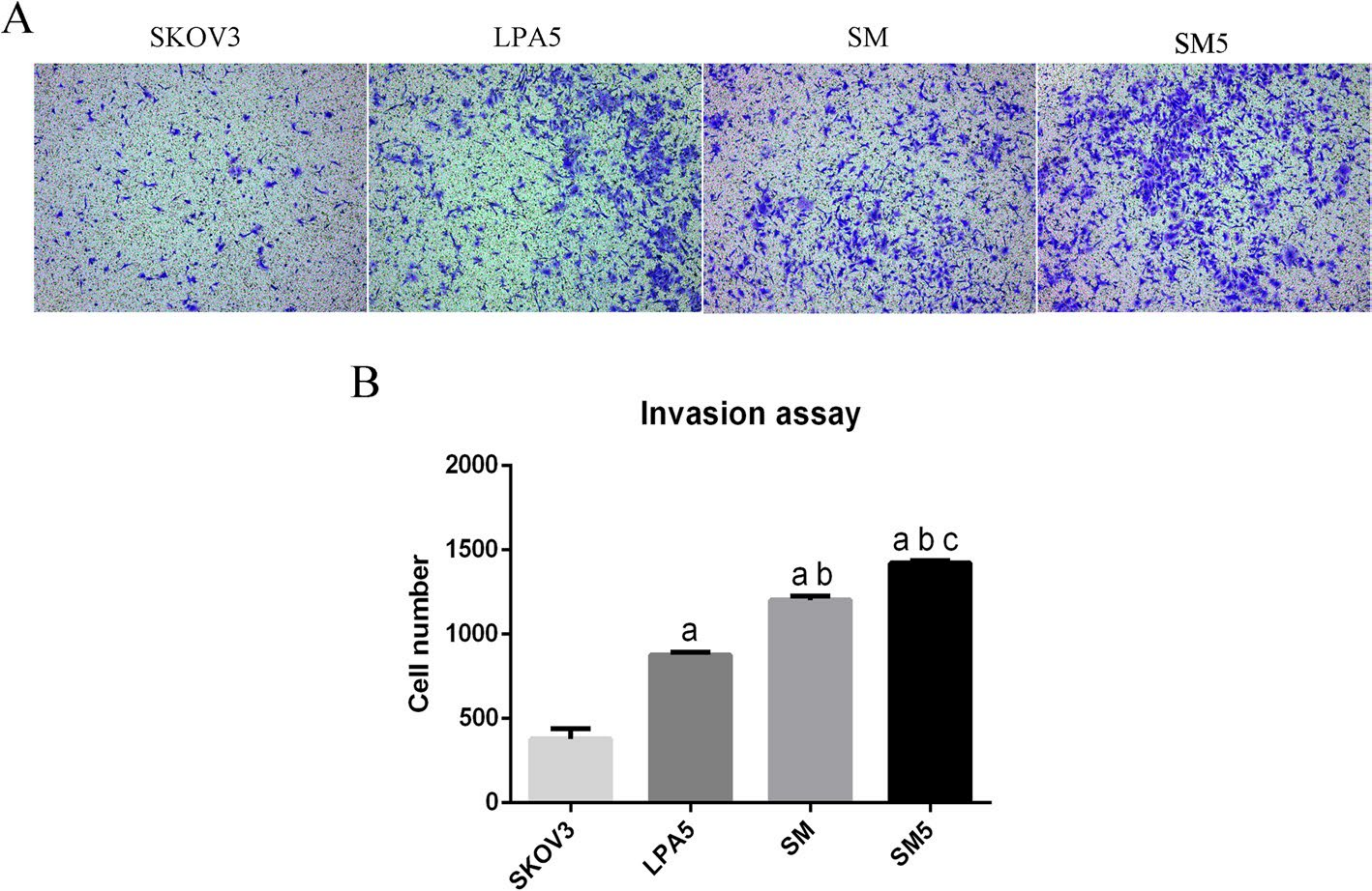
The induced monocyte-derived cells promoted the invasion of SKOV3 cells. The invasion ability of SKOV3 cells in the coculture groups (SM and SM5), especially in the SM5 group, was significantly enhanced compared with that of SKOV3 cells cultured alone (SKOV3 and LPA5). Notes: the SKOV3 cells in the SM group and SM5 group were continuously cocultured after 7 days of treatment. **a** *p* < 0.05 compared to blank; **b** *p* < 0.05 compared to LPA5; **c** *p* < 0.05 compared to MS

The invasion of SKOV3 cells was examined next. Invasion was evaluated by using 8-μm Transwell inserts and Matrigel according to the protocol and instructions. Here, the SKOV3 cells in the SM group and SM5 group were continuously cocultured after 7 days of treatment. The invasion ability of SKOV3 cells in the coculture groups, especially in the SM5 group, was significantly enhanced compared with that of SKOV3 cells cultured alone (Fig. 5).

SKOV3 cells, a human ovarian adenocarcinoma, showed increased proliferation and increased invasion after coculture with monocytes or monocytes plus LPA, further proving that these induced monocyte-derived cells were TAMs. This result could also indicate that in vivo, TAMs could exert a similar stimulatory effect on ovarian cancer cells to promote tumor progression.

### The cytokine IL-6sR and the Janus kinase/signal transducer and activator of transcription (JAK-STAT) signaling pathway played an important role in the differentiation of monocytes into TAMs

**Fig. 6:**
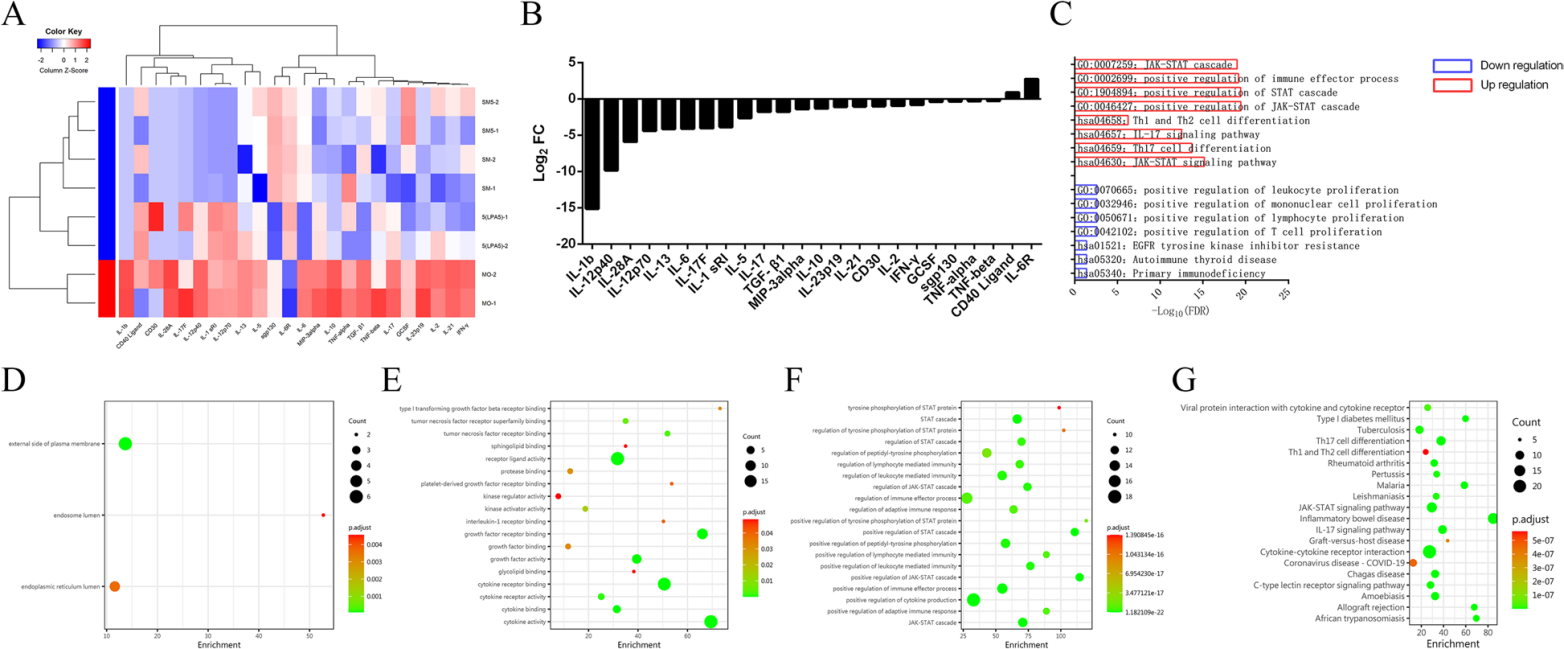
The cytokine IL-6sR and JAK-STAT signaling pathway played an important role in the differentiation of monocytes into TAMs. **A**, **B** IL-6sR was significantly elevated in the 3 treatment groups (LPA5, SM and SM5) compared to the MO group. **C**, **D**, **E**, **F** GO enrichment analysis: the main molecular functions involved were cytokine activity and growth factor receptor binding and that the main biological processes involved were positive regulation of the STAT cascade and positive regulation of the JAK-STAT cascade. **G** KEGG pathway enrichment analysis: enrichment of the JAK-STAT signaling pathway and inflammatory bowel disease pathway

The culture supernatant was collected from the four groups after 7 days of treatment. The cytokine concentrations were then assessed by EILSA array. Based on the outcomes of differential protein expression analysis, IL-6sR was significantly elevated in the 3 treatment groups (LPA5, SM and SM5) compared to the MO group (Fig. 6A, 6B). The results of GO enrichment analysis revealed that the main molecular functions involved were cytokine activity and growth factor receptor binding and that the main biological processes involved were positive regulation of the STAT cascade and positive regulation of the JAK-STAT cascade (Fig. 6C-F). KEGG pathway enrichment analysis also showed enrichment of the JAK-STAT signaling pathway and inflammatory bowel disease pathway (Fig. 6G). These results indicate that IL-6sR and the JAK-STAT signaling pathway play an important role in the differentiation of monocytes into TAMs.

## Discussion

The TME is the environment surrounding the tumor and includes immune cells, signaling molecules, ECM, blood vessels and fibroblasts [32–34]. TAMs, a type of immune cell in the TME, link inflammation with cancer and affect the behavior of tumor cells [35–38]. TAMs are associated with worse survival and prognosis in ovarian carcinoma [39]. They affect many aspects of ovarian cancer, including tumor progression, prognosis, drug resistance and immunosuppression [8]. Serous carcinoma is the most common pathological type of epithelial ovarian carcinoma [3, 40]. SKOV3 is an ovarian carcinoma cell line derived from a patient with serous ovarian cystadenocarcinoma [41]. In this study, SKOV3 cells and primary monocytes were cocultured in a system with 0.4-µm pore size Transwell chambers; thus, the transport, diffusion, secretion and communication of cytokines and other molecules were not affected, providing cells with a microenvironment similar to that found in vivo.

TAMs have an M2-like phenotype. CD163 and CD206 have been suggested to be highly expressed on TAMs in ovarian carcinoma and can be used to predict its recurrence [15, 17, 18, 42]. In addition, the LPA level is high in the blood, tissues and ascites of patients with ovarian cancer; thus, LPA is a potential marker for ovarian carcinoma [23–25]. A recent study suggested that murine macrophages differentiated from LPA-induced monocytes express CD14, CD64, CD68 and CD206, where the expression of CD163 was not evaluated in that study [30]. In this research, human monocyte-derived cells cocultured with SKOV3 cells and/or stimulated with LPA showed adherent cell growth, an increased size, an irregular shape, pseudopodia and protrusions, and positive expression of CD163, CD206, CD14 and CD163, indicating differentiation of monocytes into CD163 + macrophages (M2-like TAMs) in the simulated serous ovarian cancer microenvironment. Therefore, a similar phenomenon may also occur in the in vivo serous ovarian cancer microenvironment in humans.

TAMs have been reported to affect biological behaviors of cancer cells, including proliferation and invasion [37, 38]. In the current study, SKOV3 cells showed increased proliferation and invasion abilities after coculture with monocytes or monocytes plus LPA, especially after continuous coculture. These findings also inversely prove that the monocyte-derived cells were TAMs and that this coculture environment greatly influences tumor cell proliferation and invasion. The effect of the TME on the differentiation of monocytes into TAMs and the proliferation and invasion of ovarian cancer cells cannot be underestimated. These findings could also indicate that in vivo, TAMs may exert a similar stimulatory effect on ovarian cancer cells to promote tumor progression.

Cytokines and related signaling pathways play a vital role in tumor immunity and cell differentiation. IL-6R, also called CD126, is a type I cytokine receptor. IL-6R has two forms, IL-6sR and membrane IL-6R (IL-6mR) [43–46] IL-6sR is an agonist of IL-6 activity [47]. IL-6sR is responsible for the proinflammatory nature of IL6 and is an important participant in the development of chronic inflammatory diseases [47]. Binding of IL6 to IL-6sR is necessary to induce VEGF production [48]. The IL-6sR:IL-6 complex binds to IL-6ST/gp130 on the cell surface and induces signal transduction, even in cells that do not express IL-6mR, a process called trans-signaling [49, 50]. Then, the JAK-STAT signaling pathway is activated, [49–52] which promotes the biological functions of differentiation, proliferation, immune regulation, oxidative stress responses, etc. [53] In this study, we found that the IL-6sR and the JAK-STAT signaling pathway were significantly upregulated under conditions of monocyte coculture with SKOV3 cells and/or stimulation with LPA. IL-6sR and the JAK-STAT signaling pathway appear to played a prominent role in the differentiation of monocytes into TAMs. However, their specific role and mechanism in this process need to be further studied.

Macrophages are derived from circulating monocytes or yolk sac progenitor cells, but the exact origin of TAMs remains controversial [54]. However, macrophages at disease sites have been reported to originate primarily from the differentiation of circulating monocytes [22]. The type of macrophages in the TME depends on the type, location, and nature of the tumor, meaning that TAMs are tissue-specific and tumor-specific [55]. The differentiation of circulating monocytes in ovarian cancer patients after arriving at sites of ovarian cancer tissue remains incompletely understood. In the microenvironment established in this study, which was similar to that in vivo, monocytes differentiated into M2-like TAMs when cocultured with SKOV3 cells. This finding indicates that circulating monocytes can directly differentiate into TAMs after arriving at sites of serous ovarian cancer, and that TAMs in serous ovarian carcinoma tissue can be derived from circulating monocytes.

## Conclusions

In summary, this study demonstrated the following: the morphology and function of induced human monocyte-derived cells under conditions of coculture with SKOV3 cells and/or LPA stimulation were significantly changed compared with those of non-induced monocytes; the induced cells exhibited a CD14 + CD163 + and CD206 + phenotype, indicating that they were TAMs; the induced cells promoted the proliferation and invasion of SKOV3 cells, further proving that these cells were TAMs; and the cytokine IL-6sR and the JAK-STAT signaling pathway played an important role in the differentiation of monocytes into TAMs. Therefore, the following conclusions can be made: human monocytes can be differentiated into TAMs by coculture with SKOV3 cells and/or stimulation with LPA, monocytes in the blood can directly differentiate into TAMs after arriving at sites of serous ovarian cancer tissue, and TAMs in serous ovarian carcinoma tissue can be derived from circulating monocytes. The level of the cytokine IL-6sR was significantly increased in the medium, but the specific role and mechanism of IL-6sR and the JAK-STAT signaling pathway in this process need to be further studied.

### Materials and methods

The experimental flowchart shown in Fig. 1 gives an overview of this study. The details are as follows.

### Cell culture and ethics statement

Isolation of primary human monocytes is a core technique used in this research. The specific method was described in our previously published article [56]. Umbilical cord blood from healthy full-term deliveries (50 ml) was diluted with PBS at a ratio of 1:1, pipetted slowly onto Ficoll Paque Plus (TBD, Tianjin, China) at a ratio of 1:1, centrifuged at 1000 × g for 30 min at room temperature (acceleration and deceleration were set at zero), and peripheral blood mononuclear cells (PBMCs) were collected. Then, monocytes were isolated from PBMCs by using magnetic-activated cell sorting (MACS) with CD14 + microbeads (Miltenyi Biotec, USA) and were cultured in RPMI-1640 medium (Gibco, USA) containing 10% fetal bovine serum (FBS, Gibco) and 1% penicillin–streptomycin. The purity of the isolated cells was greater than 95%. This study was approved by the Ethics Committee of Beijing Chaoyang Hospital, Capital Medical University. Written informed consent was obtained from all patients before surgery.

The SKOV3 cell line was obtained from the Medical Research Center of Beijing Chaoyang Hospital, Capital Medical University. The use of the cell line was approved by the ethics and institutional review board. Cells were grown in RPMI-1640 medium containing 10% FBS under standard conditions (5% CO_2_, 37 °C).

### Coculture system and LPA stimulation

The coculture system was established by inserting 0.4-μm pore size Transwell chambers (Corning, USA) in 6-well plates, where cells could not pass through the membrane and the exchange of cytokines was not affected, to simulate the in vivo microenvironment of serous ovarian cancer. In this system, when monocytes were the focus, monocytes (1 × 10^6^) were cultured in the lower chamber, and SKOV3 cells (5 × 10^4^) were cultured in the upper chamber. When harvesting of SKOV3 cells was required, monocytes were cultured in the upper chamber, and SKOV3 cells were cultured in the lower chamber. Cells were cultured in RPMI-1640 medium containing 10% FBS and 1% penicillin–streptomycin.

LPA (18:1) (Avanti Polar Lipids, USA) was solubilized, and a stock solution was prepared according to the method provided in the instructions from the manufacturer. Liquid LPA was added to the culture medium to study its effect on monocytes. Preliminary experimental results showed that 5 μM LPA was the optimal concentration.

Accordingly, four groups were established: 1) MO: monocytes were cultured alone in LPA-free medium; 2) LPA5: monocytes were cultured alone in medium with 5 μM LPA; 3) SM: monocytes were cocultured with SKOV3 cells in LPA-free medium; and 4) SM5: monocytes were cocultured with SKOV3 cells in medium with 5 μM LPA.

Cells were visualized with a microscope (Olympus, CX-14, Japan) every day. The morphology of monocyte-derived cells changed significantly after 7 days. The duration of 7 days was also determined by a previous literature report [30].

### Flow cytometry

Monocyte-derived cells were harvested after 7 days of treatments. These cells were washed and stained with APC-CD14 (Biosciences, USA), PE-CD163 (Biosciences, USA), and BV-421-CD206 antibodies (Biosciences, USA) and were then analyzed by flow cytometry (FACSCanto II, BD Biosciences, USA) and FlowJo software (version 10; Tree Star, USA) was used to analyze the outcomes.

### Cell proliferation assay

SKOV3 cells were cultured in the lower chamber and monocytes were cultured in the upper chamber in the same coculture system. SKOV3 cells were harvested after 7 days. The proliferation of SKOV3 cells were evaluated by using a CCK8 kit (KeyGEN Bio TECH, China). Cell densities were calculated, and cells (3 × 10^3^) were seeded in 96-well plates. Cell viability and growth were evaluated after culture for 0 h, 24 h, 48 h and 72 h. The optical density (OD) values at 450 nm were measured in a microplate reader. “Coculture” indicates that SKOV3cells in the SM group and SM5 group were in continuous coculture after 7 days of treatment. “Monoculture” indicates that SKOV3 cells in the SM group and SM5 group were cultured alone after 7 days of treatment.

### Cell invasion assay

SKOV3 cells were cultured in the lower chamber and monocytes were cultured in the upper chamber in the coculture system. SKOV3 cells were harvested after seven days of treatment. The invasion of SKOV3 cells was evaluated by using 8-μm Transwell inserts (Corning, USA) and Matrigel (BD Bioscience, USA) according to the protocols and instructions. The membranes of the upper chambers were coated with 50 g/ml Matrigel. Cells (4 × 10^4^) suspended in medium containing 0.1% FBS were seeded in the upper chambers. Complete medium (10% FBS) was added to the lower chambers. After 24 h, the cells on the polycarbonate membranes were fixed with paraformaldehyde and stained with crystal violet. Those cells on the upper surface of membranes were wiped off with cotton-tipped applicators. Blue-stained cells refer to cells attached to the lower chamber side of the membrane after passing through the Matrigel and membrane. The invasion ability was calculated by counting the number of blue-stained cells entering the lower chamber with ImageJ software. Based on outcomes of the proliferation experiment, the SKOV3 cells in the SM group and SM5 group were continuously cocultured after 7 days of treatment in this experiment.

### Assessment of cytokines in the cell supernatant

The cell culture supernatant was collected after 7 days of treatment and stored at − 80 °C for subsequent evaluation. The concentrations of 34 Th1-, Th2- and Th17–related cytokines were detected by a RayBio® Human Th1/Th2/Th17 Antibody Array (G-Series, RayBiotech, USA).The array contained the following cytokines: CD30, CD40 ligand, CD40, granulocyte colony-stimulating factor (G-CSF), glucocorticoid-induced TNFR-related protein (GITR), granulocyte–macrophage colony-stimulating factor (GM-CSF), IFN-gamma, interleukin (IL)-1 sRI, IL-1 sRII, IL-10, IL-12 p40, IL-12 p70, IL-13, IL-17A, IL-17F, IL17R, IL-1β, IL-2, IL-21, IL-21R, IL-22, IL-23p19, IL-28A, IL-4, IL-5, IL-6, soluble IL-6 receptor (IL-6sR), macrophage inflammatory protein (MIP)-3α, sgp130, transforming growth factor (TGF)-β1, TGF-β3, TGF-α, TGF-β and TNF-related activation-induced cytokine (TRANCE). The images and signals were aquired using a GenePix 4000B scanner (Molecular Devices, LLC, USA). RayBio® Analysis Tool software was used to analyze the data. Subsequently, differential protein expression analysis, gene ontology (GO) enrichment analysis (including molecular function (MF), biological process (BP) and cellular component (CC) terms) and KEGG pathway enrichment analysis were performed.

### Statistical analysis

Data were collected from at least 3 independent experiments. SPSS 23.0 (IBM Corp., USA) and GraphPad Prism software (GraphPad, Inc., USA) were used for statistical analysis. A t-test and ANOVA were used to evaluate differences between groups. Quantitative data are shown as the mean ± SEM values. Statistical significance was defined as a two-sided p-value of less than 0.05.

## Data Availability

All data generated or analysed during this study are included in this published article.
